# Which methods to choose to correct cell types in genome-scale blood-derived DNA methylation data?

**DOI:** 10.1186/1471-2105-16-S15-P7

**Published:** 2015-10-23

**Authors:** Akhilesh Kaushal, Hongmei Zhang, Wilfried JJ Karmaus, Julie SL Wang

**Affiliations:** 1 Division of Epidemiology, Biostatistics, and Environmental Health, University of Memphis, Memphis, TN 38152, USA; 2 Division of Environmental Health & Occupational Medicine, National Health Research Institutes, Miaoli, 360, Taiwan

## Background

High throughput methods such as microarray and DNA-methylation are used to measure the transcriptional variation due to exposures, treatments, phenotypes or clinical outcomes in whole blood, which could be confounded by the cellular heterogeneity[1,2]. Several algorithms have been developed to measure this cellular heterogeneity. However, it is unknown whether these approaches are consistent, and if not, which method(s) perform better.

## Materials and methods

The data implemented in this study were from a Taiwan Maternal and Infant Cohort Study[3,4]. We compared five cell-type correction methods, including four methods recently proposed: the method implemented in the minfi R package[5], the method by Houseman et al.[6], FaST-LMM-EWASher[7], RefFreeEWAS[8]) and one method using surrogate variables[9] (SVAs). The association of DNA methylation at each CpG site across the whole genome with maternal arsenic exposure levels was assessed adjusting for the estimated cell-types. To further demonstrate and evaluate the methods that do not require reference cell types, we first simulated DNA methylation data at 150 CpG sites across 600 samples based on an association of DNA methylation with a variable of interest (e.g., level of arsenic exposure) and a set of latent variables representing “cell types”. We then simulated DNA methylation at additional CpG sites only showing association with the latent variables.

## Results

**Figure 1:**
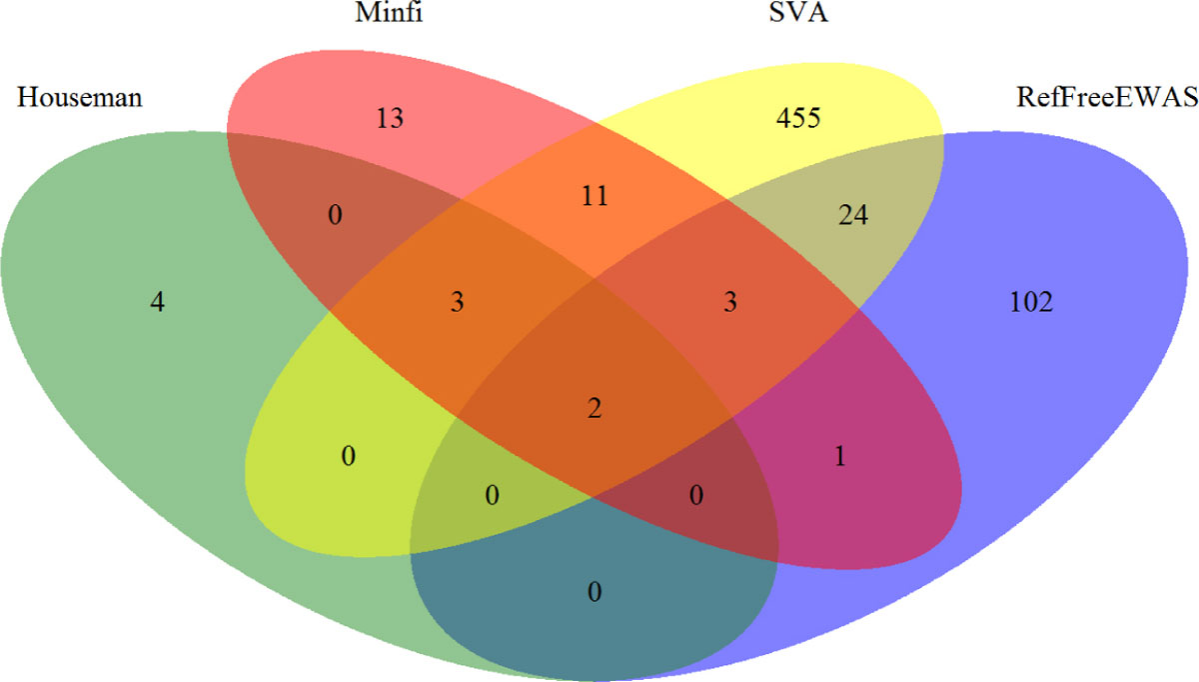
Venn diagram illustrating the overlap of significant CpG sites at FDR level of 0.05 after adjusting for cell types by different methods for the association study of maternal arsenic exposure with DNA-methylation.

Only 3 CpG sites showed significant associations with maternal arsenic exposure at a false discovery rate (FDR) level of 0.05, without adjusting for cell types. Adjustment by FaST-LMM-EWASher did not identify any CpG sites. For other methods, Figure 1 illustrates the overlap of identified CpG sites. Further simulation studies on methods free of reference data (i.e., FaST-LMM-EWASher, RefFreeEWAS, and SVA) revealed that RefFreeEWAS and SVA provided good and comparable sensitivities and specificities, and FaST-LMM-EWASher gave the lowest sensitivity but highest specificity (Table 1).

**Table 1:** Sensitivity and specificity with respect to truly identified variables using 100 simulated data; CI: confidence interval

	Sensitivity: Median (95% CI)	Specificity: Median (95% CI)
**FaST-LMM-EWASher**	0.00 (0.00, 0.52)	1.00 (0.99, 1.00)
**RefFreeEWAS**	0.98 (0.00, 1.00)	0.94 (0.93, 1.00)
**SVA**	1.00 (0.98, 1.00)	0.94 (0.93, 0.94)

## Conclusions

The results from real data indicated RefFreeEWAS and SVA were able to identify a large number of CpG sites, and results from SVA showed the highest agreement with all other approaches. Simulation studies further confirmed that RefFreeEWAS and SVA are comparable and perform better than FaST-LMM-EWASher. Overall, the findings support a recommendation of using SVA to adjust for cell types due to its highest agreement with other methods and appealing findings from simulation studies.
